# Colorimetric detection of both total genomic and loci-specific DNA methylation from limited DNA inputs

**DOI:** 10.1186/s13148-015-0100-6

**Published:** 2015-07-11

**Authors:** Eugene J. H. Wee, Thu Ha Ngo, Matt Trau

**Affiliations:** Centre for Personalized NanoMedicine, Australian Institute for Bioengineering and Nanotechnology (AIBN), The University of Queensland, St Lucia, Queensland Australia; School of Chemistry and Molecular Biosciences, The University of Queensland, St Lucia, Queensland Australia; Faculty of Biotechnology, Vietnam National University of Agriculture, Hanoi, Vietnam

**Keywords:** MBD enrichment, DNA methylation, Colorimetric, Electrochemical, GSTP1

## Abstract

**Background:**

Aberrant DNA methylation marks are potential disease biomarkers, and detecting both total genomic and gene-specific DNA methylation can aid in clinical decisions. While a plethora of methods exist in research, simpler, more convenient alternatives are needed to enhance both routine diagnostics and research.

**Results:**

Herein, we describe colorimetric assays using methyl-binding domain (MBD) proteins for rapid and convenient evaluation of total genomic and gene-specific methylation from 50 ng or less DNA input in under 2 h. As little as 5 % methylation differences can be detected and are enhanced by a novel MBD protocol for improved specificity. Our assays could differentiate naïve from de-methylating drug-treated cells and detect the presence of a methylated prostate cancer biomarker in the urine. Finally, the assay was evolved onto disposable screen-printed electrodes for convenient detection of gene-specific methylation in urine.

**Conclusions:**

Rapid MBD-based colorimetric and electrochemical approaches to detect DNA methylation from limited samples were successfully demonstrated and applied to clinical samples. We envision that the ease, low sample requirements and speed of these assays could have both clinical and research-wide applications.

**Electronic supplementary material:**

The online version of this article (doi:10.1186/s13148-015-0100-6) contains supplementary material, which is available to authorized users.

## Background

Aberrant epigenetic changes in DNA are potential disease biomarkers [1–3]. A form of epigenetic change is the methylation of the cytosine (5mC) in cytosine/guanine dinucleotides (CpG), particularly in CpG islands (CGI) of regulatory regions that function to regulate cellular processes [1–3]. Azanucleoside drugs such as 5-aza-2′-deoxycytidine (5-Aza) have been used therapeutically with some success to reactivate silenced genes in epigenetic diseases [4–6]. In addition, genome-wide hypomethylation is also associated with tumorigenesis [2, 3] and hence may be useful as an early screening strategy for cancer. While hypomethylation is associated with tumorigenesis, regulatory sequences at specific loci, such as that of tumour suppressor genes, are hypermethylated and detection of which are potentially useful in stratifying patient cohorts and informing clinical decisions [1–3, 7]. Most approaches, however, detect DNA methylation via bisulfite conversion [8, 9] of DNA followed by some form of sequencing [10–13]. To avoid the problems associated with bisulfite conversion, affinity capture approaches, such as methyl-binding domain (MBD) proteins or antibodies raised against 5mC, have been adapted to Next Generation Sequencing [14] platforms or other optical detection methods for both genome-wide [15, 16] and gene-specific [17] applications. Useful, simpler, more convenient methods to detect both genome-wide and gene-specific methylation are still lacking and would be useful for both routine diagnostics and research.

MBD enrichment approaches are useful and convenient because they avoid the limitations of bisulfite conversion while being very highly specific for 5mC on native double-stranded DNA but not hydroxymethylated (5hmC) or unmethylated DNA [18]. Unfortunately, MBD enrichment approaches are limited by their difficulty in quantifying methylation levels and typically quantitative PCR (or sequencing) is used to measure enrichment levels as a proxy estimate of differential methylation [19]. Additionally, the stringency of MBD enrichment reduces with limiting DNA inputs and various strategies including high-salt buffers [19] and alternative MBD enzymes [20] have been devised. Nonetheless, one is still able to infer, with very high stringency, the degree/density of methylation based on the buffer conditions required to recover enriched DNA [14, 19]. In short, the methylation outcomes derived from MBD approaches are generally binary, i.e., yes/no calls and therefore, ideal for identifying highly differentially methylated regions (HDMRs).

Colorimetric readouts are also popular in molecular diagnostics because they can be evaluated with the naked eye and have the option for (semi)quantification. One popular colorimetric system is the horse radish peroxidase HRP/H_2_O_2_ system coupled to a chromogen e.g., 3,3′,5,5′-tetramethylbenzidine (TMB) substrate to generate a coloured by-product to signal the presence of a biomolecule, (HDMRs in this case). Herein, we describe MBD-based colorimetric assays for rapid, naked-eye evaluation of either overall genome-wide or gene-specific methylation detection. In addition, we also describe a novel method for rapid yet highly specific MBD enrichment from low nanogram amounts of DNA input for loci-specific applications that was subsequently applied to urine DNA derived from prostate cancer patients. Finally, since TMB is electrochemically active [21], we adapted our assay onto commercially available screen-printed electrodes as a potential alternative electrochemical approach for detecting DNA methylation. To the best of our knowledge, these are the first demonstrations of colorimetric and electrochemical evaluations of DNA methylation via MBD and may have wide applications in both research and diagnostics.

## Results and discussion

### The MBD/HRP assays

**Fig. 1:**
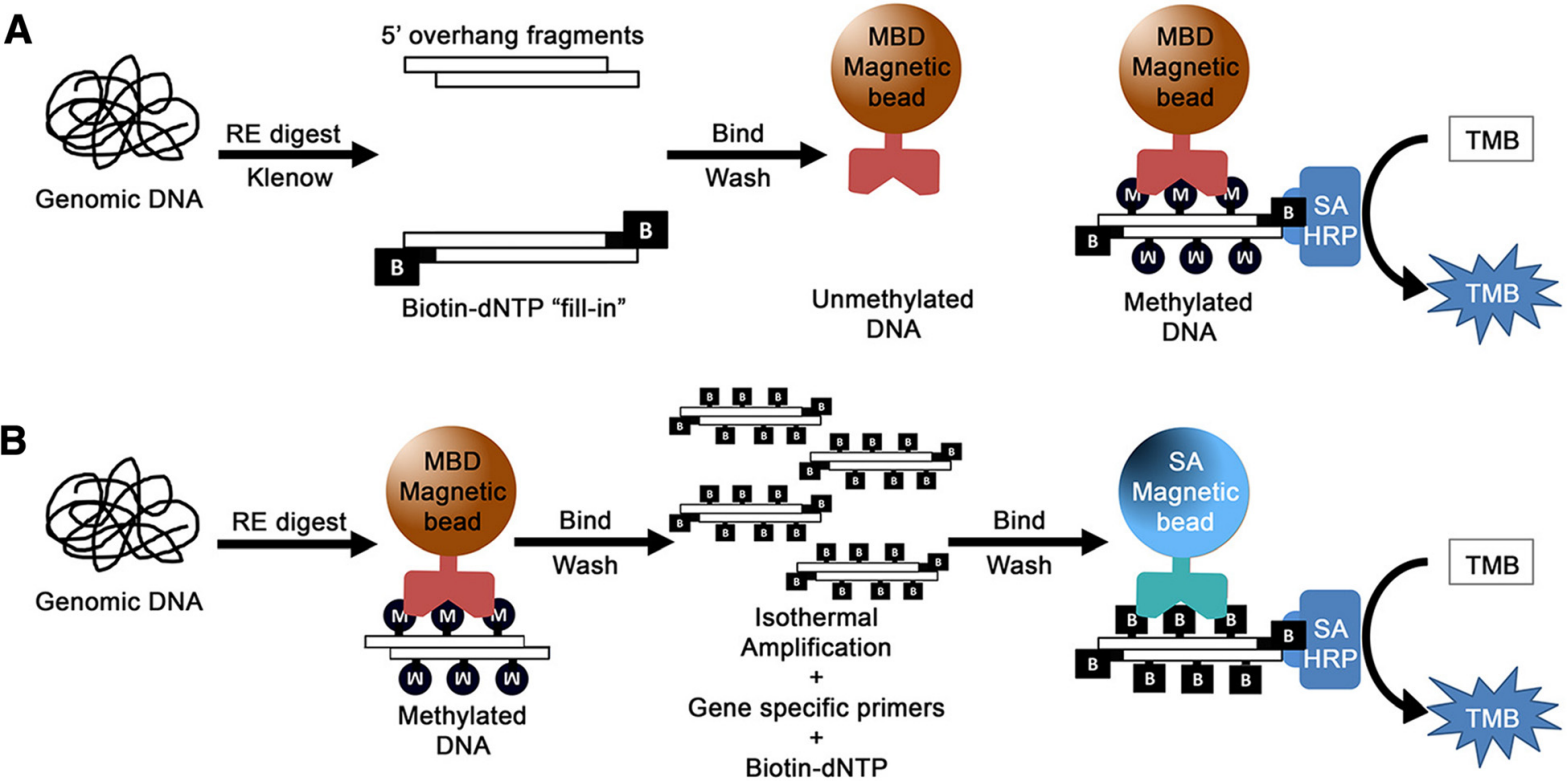
Assay schemes. **a** Strategy for total genomic methylation. Genomic DNA is restriction enzyme (*RE*) digested, enzymatically biotinylated via a fill-in reaction with Klenow polymerase and biotin-dNTPs. MBD magnetic beads are then used to select for methylated DNA. Colorimetric evaluation is mediated by SA-HRP which recognizes the biotin on enriched methylated DNA. **b** Strategy for gene-specific methylation. Genomic DNA is RE digested and methylated DNA is selected via MBD enrichment. Gene-specific isothermal amplification is then performed with biotin-dNTP to generate biotin-DNA polymers which are in turn selected for with SA magnetic beads and SA-HRP for colorimetric evaluation

To realize a simple MBD-based approach for naked-eye detection of DNA methylation, we first developed a proof-of-concept method for overall genomic methylation (Fig. 1a). Then, based on a similar strategy, the assay was extended to loci-specific targets (Fig. 1b). Both approaches began with an enzymatic digestion of genomic DNA (gDNA) to fragment and reduce the complexity of gDNA for optimal MBD enrichment. For genome-wide analysis (Fig. 1a), digested gDNA fragments were then enzymatically labelled with biotin with a “fill-in” reaction to generate a DNA/biotin polymer (see Methods for detailed procedure). MBD2a (a member of the MBD protein family) that had been conjugated to a paramagnetic particles was then used to select for methylated DNA. The enriched biotin-labelled methylated DNA was, in turn, recognized by streptavidin-conjugated horse radish peroxidase (SA-HRP) via the biotin/streptavidin interaction. Finally, methylation levels were then visually evaluated via the HRP-mediated reduction of a chromophore (e.g., TMB substrate). The intensity of the developed colour was proportional to the amount of captured DNA and thus the level of methylation.

To enable gene-specific applications (Fig. 1b), digested DNA was first subjected to a novel protocol for rapid yet highly specific MBD enrichment from limiting samples (details in a later section). This was then followed with an isothermal amplification of the desired region with biotin-deoxynucleotides (dNTPs) to generate polymers of biotin/DNA which then served as substrates for the HRP/TMB colorimetric reaction and therefore indicating the presence of a HDMR.

### Detecting genome-wide methylation

**Fig. 2:**
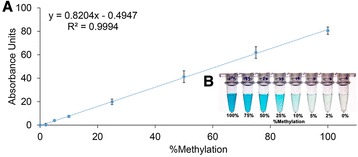
Total methylation assay performance. **a** Calibration plot of HRP/TMB colorimetric response with respect to changes in amount of methylated DNA. *Error bars* represent SD, *n* = 3. **b** Photo of HRP/TMB reactions used to generate the calibration plot

To demonstrate the feasibility of genome-wide methylation assay, we first tested if we could detect 200 pg of 119-bp synthetic sequence at various levels of methylation (Fig. 2). Figure 2a shows the positive relationship between methylation and chromophore intensity (measured as absorbance at 650 nm). Near baseline signals from the 0 % methylated sample indicates the high specificity of the assay. Consistent with the literature [18], the MBD assay was also insensitive to hydroxymethylated DNA under the current assay conditions (Additional file 1: Figure S2). To determine the sensitivity of the assay i.e., the smallest detectable change in percent methylation, we compared the signal generated at 2, 5 and 10 % methylation with that of the 0 % methylated sample. Since signal at 5 % was significantly higher (*t* test *p* < 0.05), we concluded that the assay was sensitive to at least 5 % methylation changes. In contrast, 10 % or more methylated samples are easily differentiated by eye from the unmethylated control (Fig. 2b). Similar results were also seen with in vitro methylated whole genome amplified DNA (M-WGA, Additional file 1: Figure S1) where as little as 37.5 pg of methylated DNA could be detected. In contrast, current commercial ELISA-based approaches advertise a 200-pg or more detection limit. Finally, we evaluated the stability of the assay (Additional file 1: Figure S2) and found that the inter- and intra-assay coefficient of variability (CV) were 7.9 % (*n* = 3) and 5.2 % (*n* = 6), respectively, indicating good reproducibility.

While we demonstrate a wide dynamic range of methylation in this study, the method may be tuned to narrower ranges by adjusting the buffer conditions to detect meaningful levels of methylation over normal levels. However, given the current limits in detection and reproducibility, the assay would more likely be suited to detect differential methylation changes of 10 % or more in a clinical setting.

Total DNA methylation is traditionally detected by HPLC [22] and mass spectrometry (MS) [23] methods. While our MBD assay uses less starting material, it is difficult to directly compare the methylation-based analytical performances. This is because both HPLC and MS approaches consider all cytosines in the genome whereas our assay is an enrichment approach that only considers a subset of cytosines and therefore a different measure of methylation. Nonetheless, compared to the published data in a previous MBD/flow cytometry study [16], the current approach had similar analytical performance but only required a fraction of the processing time (2 versus 24 h). Finally, in comparison to the published data in a recent study via a proximity oxygen channelling chemistry platform [15], both approaches had similar performance in detecting methylated DNA. However, the oxygen channelling platform requires very specialized equipment for detection whereas the colorimetric strategy described herein does not.

**Fig. 3:**
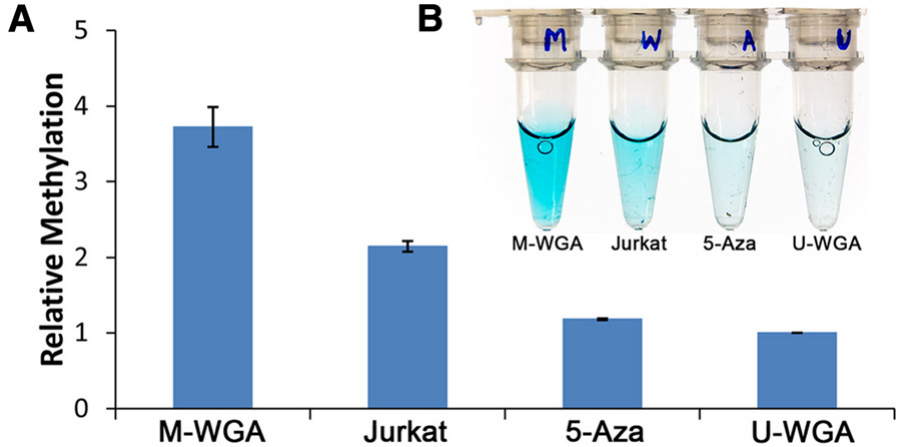
Differentiating between 5-Aza-treated and untreated cells. **a** Colorimetric response of highly methylated M-WGA DNA, naïve Jurkat cells and Jurkat cells after 5-Aza treatment relative to unmethylated U-WGA control. *Error bars* represent SD, *n* = 3. **b** Photo of the colorimetric assay from the corresponding experiments

Next, we sought to demonstrate a potential clinical application in tracking response to de-methylating therapy. To this end, the assay was performed using genomic DNA derived from human cancer cell lines before and after 5-Aza treatment and with M-WGA and unmethylated WGA DNA (U-WGA) as controls (Fig. 3). We could clearly distinguish between M-WGA and U-WGA and, as expected, differentiate DNA samples before and after de-methylating treatment. Traditional approaches, such as HPLC and MS, while useful, have difficult sample preparation and require high amounts of input DNA (micrograms) [24] thus severely limiting its adoption in routine diagnostics. In contrast, our approach required at least one order of magnitude lesser DNA (~50 ng) of starting material, had similar performance to commercial ELISA-based assay and required approximately 2 h to complete.

### Improved MBD protocol for highly specific enrichment from limited DNA input

A potential limitation of MBD enrichment strategies is non-specific capture of unmethylated DNA. This problem is especially pronounced with low DNA input because of increased background from non-specifically captured unmethylated DNA and is further confounded when coupled to an exponential DNA amplification for gene-specific applications. Various strategies to alleviate this problem include multiple methylation-sensitive restriction digestion [25], exotic MBD complexes [20], high-salt-binding buffers and incubation at low temperatures [19]. While these approaches improved specificity, their long overnight incubation may not be feasible for applications requiring fast turnovers e.g., routine diagnostics.

Nonetheless, the results of these studies suggested that when MBD was in excess, as in the case of low sample input, MBD non-selectively bound both methylated and unmethylated DNA. Hence, we hypothesized that increasing the DNA to MBD ratio could restore the selectivity of MBD for methylated DNA. One possible way to increase the DNA/MBD ratio is to introduce exogenous carrier/blocking DNA, such as salmon sperm DNA, to sequester free MBD.

**Fig. 4:**
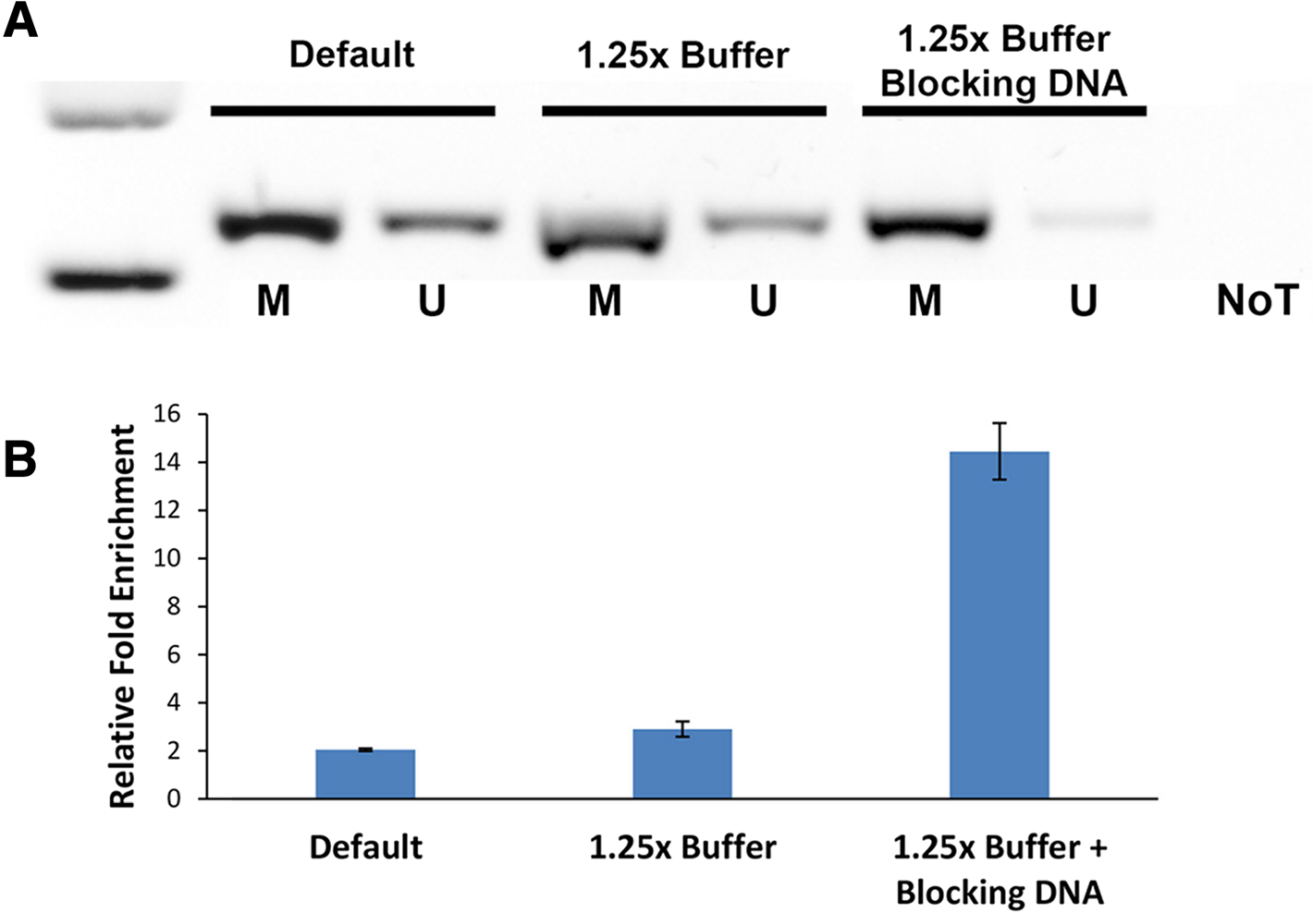
Improved MBD enrichment. **a** Gel electrophoresis image of RPA amplifications after MBD enrichment under the default manufacturer’s protocol, increased salt with 1.25× buffer, and with 1.25× buffer supplemented with 50 ng of blocking DNA. **b** Graph showing the relative improvement in enrichment. *Error bars* represent SD, *n* = 3. *M* M-WGA, *U* U-WGA, *NoT* no template control

To this end, we arbitrary added 50 ng of salmon sperm DNA to 50 ng of human WGA DNA and performed the MBD enrichment. Under this condition, minimal non-specific enrichment of U-WGA was detected with minimal lost in performance for M-WGA based on subsequent isothermal amplification and electrophoresis analysis (Fig. 4), thus supporting our hypothesis. Using densitometry, the signal to noise (M-WGA and U-WGA, respectively, post-MBD enrichment) with our approach was 14.2-fold which was a marked improvement over the default manufacturer protocol (2.1-fold) or just an increase in buffer salt concentrations (3.1-fold). In short, these results confirmed our hypothesis that an increased DNA/MBD ratio could enhance specificity. To our knowledge, this is the first description of exogenous blocking DNA being used to improve stringency of MBD-based assays. In contrast to the long overnight protocols previously described in the literature, our approach required only a 15-min MBD incubation (Additional file 1: Figure S3). This rapid, simple and low-cost solution may be useful for applications where sample DNA is limited.

### Detecting GSTP1 methylation with the MBD/HRP assay

Confident of the stringency displayed by our MBD enrichment protocol, we next turned our attention to designing a methylation assay against GSTP1, a clinically important HDMR biomarker of prostate cancer [26, 27]. Currently, the methylation status of GSTP1 is the only FDA-approved methylation biomarker undergoing clinical trials [28] and is usually detected by head loop PCR [29]. While potentially useful, the approach has various limitations such as the need for bisulfite conversion and complicated primer design. Simpler assays are hence needed to facilitate widespread adoption. The MBD/HRP approach described herein may fulfil this need.

**Fig. 5:**
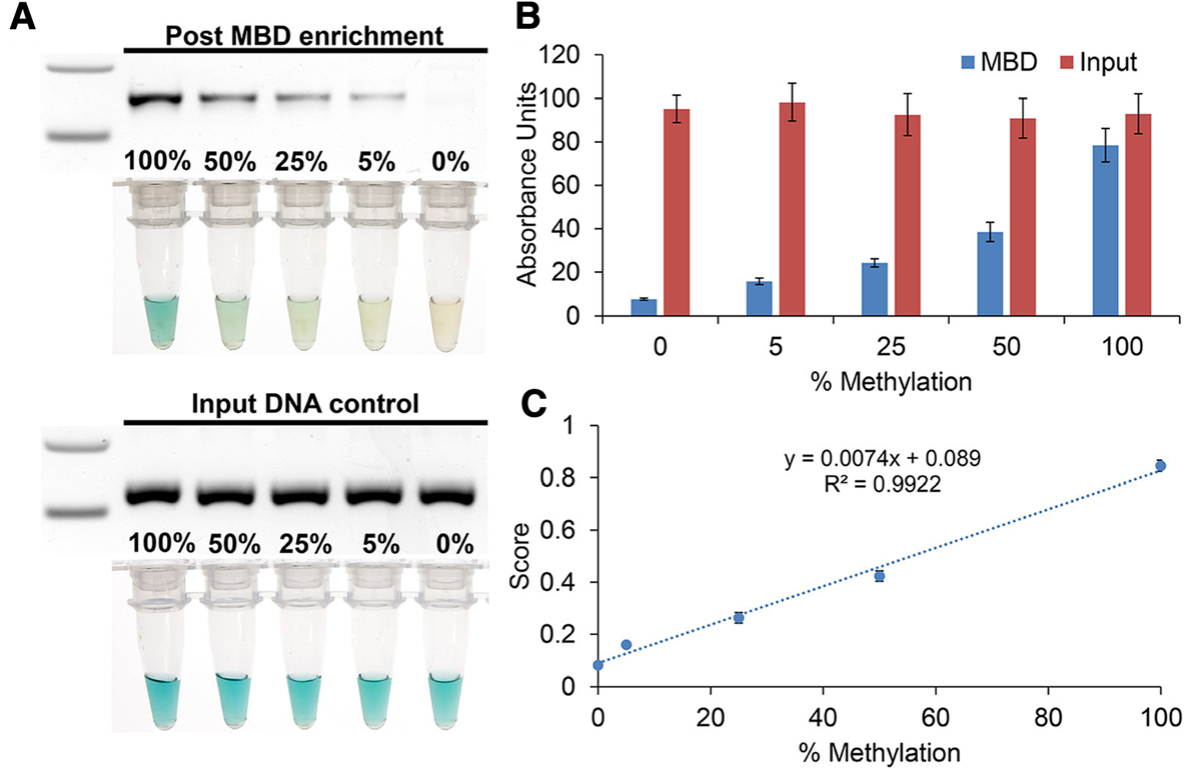
Performance of gene-specific assay for 100, 50, 25, 5, and 0 % methylated DNA samples. **a** Gel electrophoresis images of RPA amplifications and photo of HRP/TMB reactions. *Top*: post-MBD enrichment. *Bottom*: input controls i.e., before MBD enrichment. **b** Corresponding absorbance measurements. *Blue*: post-MBD enrichment. *Red*: input control. **c** Calibration plot of HRP/TMB response to methylation changes. *Error bars* represent SD, *n* = 3

As shown in Fig. 5, the assay could detect as little as a 5 % methylated sample. As a control, we also tested the input DNA prior to MBD enrichment. Since the amount of input DNA can affect the resultant amount of amplification, we therefore propose that to improve accuracy, the ratio of the signal generated post-MBD to pre-MBD (initial DNA input) be used to generate a methylation score (Fig. 5c). This method also had good reproducibility with intra-assay CV (*n* = 8) of 7.8 % and inter-assay CV (*n* = 4) of 9.1 % (Additional file 1: Figure S4).

**Fig. 6:**
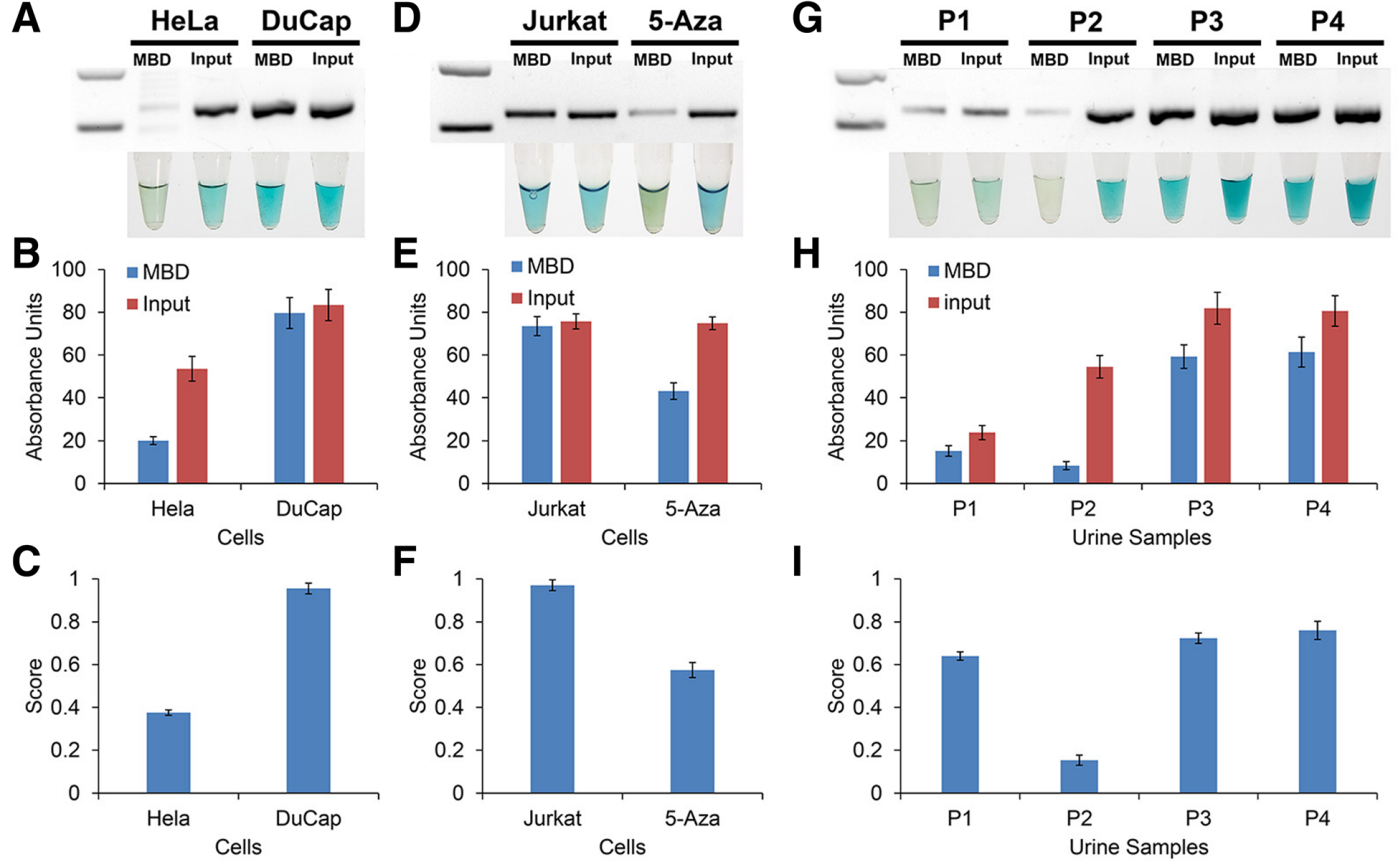
GSTP1 methylation in samples. Detecting GSTP1 methylation in cell lines (**a**–**c**), cells before and after 5-Aza treatment (**d**–**f**) and in prostate cancer patients (**g**–**i**). **a**, **d**, **g** Gel electrophoresis images of RPA amplifications and photos of HRP/TMB reactions. **b**, **e**, **h** Corresponding absorbance measurements. *Blue*: post-MBD enrichment. *Red*: input control. **c**, **f**, **i** Normalized HRP/TMB response (score) to methylation changes. *Error bars* represent SD, *n* = 3

To demonstrate feasibility on complex biological samples, we first assayed two cancer cell lines (Fig. 6a–c). DuCap cells were found to be methylated but less so for HeLa cells. We then assay Jurkat cells before and after 5-Aza treatment (Fig. 6d–f). As expected, GSTP1 was de-methylated significantly after 5-Aza treatment. Finally, the method was then applied to four DNA samples derived from the urine of metastatic, castration-resistant prostate cancer patients (Fig. 6g–i). Three of the four patients were found to be highly methylated at the GSTP1 gene promoter. Together, these results indicated that the MBD/HRP colorimetric assay may be a useful alternative for detecting GSTP1 methylation in the clinic.

The assay was further extended to the ESR1 gene promoter which is also aberrantly methylated in cancers [30]. To this end, we assayed Jurkat cells before and after 5-Aza treatment with M-WGA and U-WGA as specificity controls (Additional file 1: Figure S5). As expected, ESR1 was de-methylated in the 5-Aza-treated cells compared to naïve Jurkat cells. The de-methylation of both GSTP1 and ESR1 in 5-Aza-treated cells were consistent with the genome-wide hypomethylation observed in Fig. 3.

Based on the data shown here, a MBD/HRP-based approach may be useful for both total genomic and gene level methylation. However, a known limitation of all MBD-based approaches is the inability to provide methylation information at individual CpGs. In addition, MBD is also not able to provide 5hmC information [18]. While methods exist for 5hmC, there is no simple *single*-*step* method besides HPLC and/or MS able to simultaneously interrogate both 5mC and 5hmC. Nonetheless, this MBD/HRP approach could be adapted to an analogous assay for 5hmC using affinity reagents currently available for 5hmC [31].

### Adapting to an electrochemical assay using disposable screen-printed electrodes

**Fig. 7:**
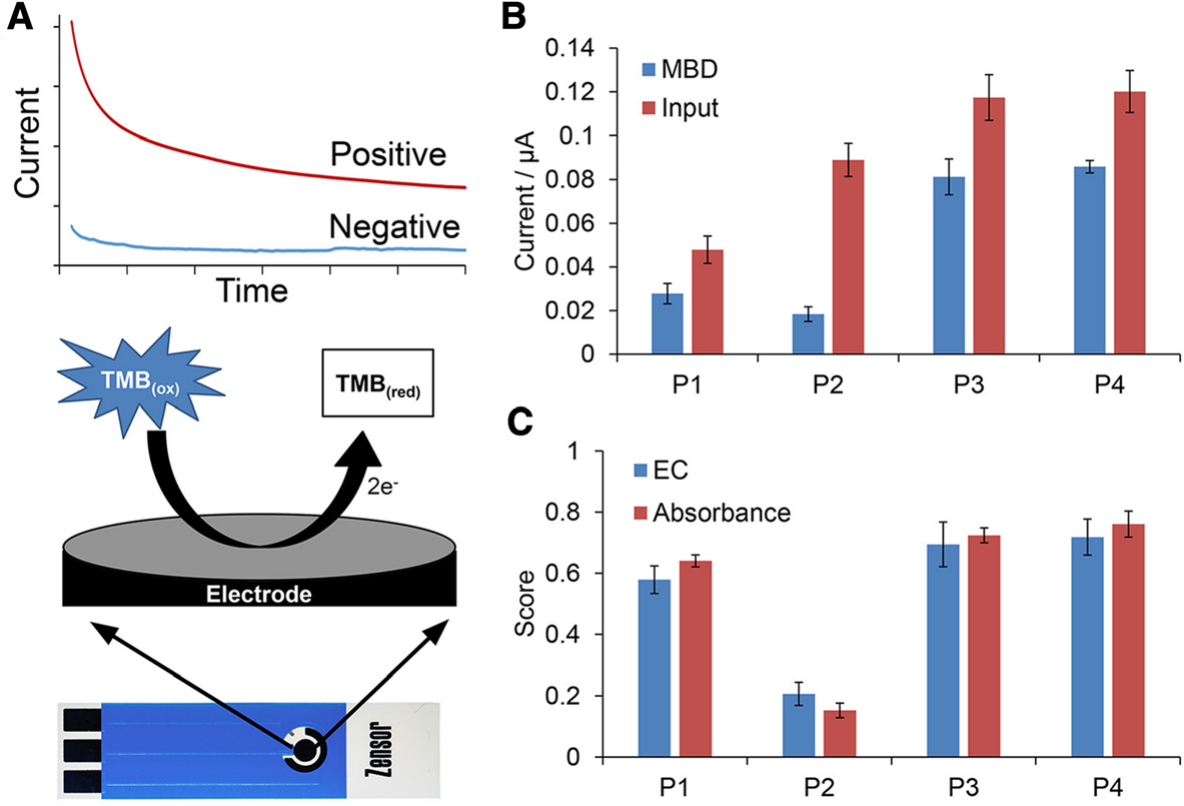
Electrochemical detection of GSTP1 methylation in urine DNA. **a** Conceptual schematic of the electrochemical detection of HRP oxidized TMB (*TMB*_*ox*_). Photo of disposable SPCE is shown. On the electrode surface, *TMB*_*ox*_ is reduced back to *TMB*_*red*_. The resulting current is proportional to the amount of *TMB*_*ox*_. **b** Current measurements of the four patient samples. *Blue*: post-MBD enrichment. *Red*: input control. **c** Comparison of methylation scores for patient samples determined electrochemically (*blue*) and by absorbance (*red*). *Error bars* represent SD, *n* = 3

The benefits of an electrochemical assay include miniaturization, improved performance and reduced cost [32–34]. Since TMB is electrochemically active [21], we had the potential to adapt our MBD/HRP assay onto commercially available screen-printed carbon electrodes (SPCE) as a convenient alternative readout platform (Fig. 7a). As a proof of concept, we applied the electrochemical assay on the same four prostate cancer samples. As expected, methylation scores were identical to that derived via spectrometry (Fig. 7b and Additional file 1: Figure S6). This not only demonstrated the feasibility of an electrochemical MBD-based assay for detecting DNA methylation in urine (and possibly other bodily fluids), it also demonstrated that our methylation assay was readout agnostic and may be adapted to other readout methods for added convenience as a diagnostic assay. To the best of your knowledge, this is also the first electrochemical assay for methylation detection via MBD.

## Conclusions

In conclusion, we have developed simple and rapid approaches to detect total genomic DNA methylation and at specific gene sequences. The assays were first developed on a colorimetric readout for naked-eye evaluation and subsequently onto electrochemical approach for convenient diagnostic and research applications. We also described a novel MBD protocol with improved stringency by exploiting the competitive binding of non-human carrier DNA to sequester excess MBD. The high sensitivity, ease and low sample requirements of our approach may be useful for routine diagnostics and a wide range of applications unlike traditional methods.

## Availability of supporting data

The data sets supporting the results of this article are included within the article and its additional files.

## Methods

### DNA preparation

To demonstrate the feasibility of the assay, a 119-bp DNA fragment model system over the GSPTP1 gene was PCR amplified using standard procedures using the Kapa2G Robust PCR kit (KapaBiosystems, USA) with forward and reverse primers (AACCCCCTTATCCCTCCGTCGTGTGGCTTTTAC and AAACAGGTTCCTCCGAAGATTTCACACAACACT, respectively (Integrated DNA Technologies, Australia). Amplicons were then purified using QIAquick Gel extraction kit (Qiagen, Australia). To generate methylated sequences, amplicons were treated with SssI methyltransferase (New England Biolabs, USA) overnight as recommended by the manufacturer and purified using the Agencourt AMPure XP kit (Beckman Coulter, Australia). An aliquot of the SssI-treated DNA was used to evaluate the methylation conversion efficiency by digestion with the methylation-sensitive HpaII restriction enzyme (New England Biolabs, USA). Only reactions with no detectable digestion via gel electrophoresis were used in downstream experiments. Finally, the amplicons were treated with 5 μM biotin-14-dUTP (Thermo Fisher, Australia) and terminal deoxynucleotidyl transferase (TdT, New England Biolabs, USA) as recommended by the manufacturer.

WGA DNA was generated using the REPLI-g UltraFast Mini kit (Qiagen, Australia) and purified using the DNeasy Blood and Tissue kit (Qiagen, Australia). An aliquot of WGA DNA was then treated with SssI methyltransferase overnight and purified to generate highly methylated genomic DNA (M-WGA). An aliquot of the SssI-treated DNA was used to evaluate the methylation conversion efficiency by digestion with the methylation-sensitive HpaII restriction enzyme (New England Biolabs, USA). Only reactions with no detectable digestion via gel electrophoresis were used in downstream experiments. Genomic DNA from Jurkat cells representing before and after 5-aza-2′-deoxycytidine treatment were purchased from New England Biolabs (NEB).

HeLa and DuCap cells were purchased from ATCC and cultured according to the manufacturer’s instructions. gDNA was extracted using the DNeasy Blood and Tissue kit. Thirty millilitres urine samples from prostate cancer patients were collected with the relevant ethics approval from The University of Queensland Institutional Human Research Ethics Committee (Approval No. 201400012) and processed within 5 h with the ZR Urine DNA Isolation Kit (Zymo Research, USA).

For total genomic methylation studies, 50 ng of gDNA (both WGA and cell line derived) was enzymatically digested with the endonucleases DpnII and MseI (7.5 units each, NEB) at 37 °C in a 20-μL reaction supplemented with the NEB Buffer 3.1 system to generate <1000 bp DNA fragments with 5′ overhangs. After 30 min, the reaction was supplemented to a final volume of 25 μL with 5 units of Klenow fragment (3′→5′ exo-) DNA polymerase (NEB, USA) and 5 μM of biotin-14-dUTP, dATP, dGTP and dCTP and incubated at 37 °C for another 30 min to fill in the overhangs and biotinylate the fragmented DNA. The reactions were then heat inactivated at 75 °C for 20 min.

For gene-specific applications, 50 ng of gDNA was digested with MseI and MluCI (NEB, USA) instead at 37 °C for 30 mins in a 20-μL reaction supplemented with the NEB CutSmart Buffer system.

### Total genomic methylation assays

For total genomic methylation studies, digested gDNA reactions were first diluted tenfold in water. One microlitre (i.e., 200 pg of DNA) was then used in the MBD/HRP assay to estimate levels of methylation. MBD-modified magnetic beads (NEB, USA) were prepared and used as recommended by the manufacturer. To enrich for methylated DNA, the provided 1× MBD buffer was supplemented with 600 mM NaCl. After a 15-min incubation with DNA targets, the MBD beads were isolated with a magnet and the supernatant was removed. The MBD beads were then resuspended in 20 μL of 1/1000 HRP solution (BD Biosciences, Australia) in 1× MBD buffer for 10 mins. The MBD beads were then washed four times with 1× MBD buffer. Finally, 100 μL of 1-Step™ TMB substrate solution (Thermo Scientific, Australia) and colorimetric changes were monitored at 650 nm over 30 min on the EnSpire® plate reader (Perkin Elmer, Australia). Absorbance values at 20 min were used in subsequent data analysis.

### Gene-specific assays

For gene-specific applications, 19 μL of digested DNA was used for MBD enrichment in a modified protocol. Briefly, one tenth of the recommended MBD/bead mix was used in a 100-μL reaction. MBD buffer (1×), MBD buffer (1.25×) or MBD buffer (1.25×) supplemented with 50 ng of salmon sperm DNA (Sigma Aldrich, Australia) was used for a 15-min MBD enrichment step at 4 °C. After three 5-min washes with 1× MBD buffer, the captured DNA was eluted in 5 μL 2.5 M NaCl solution. To purify the enriched DNA for downstream amplification, 10 μL of Agencourt AMPure XP bead solution was added to the same tube and purification proceeded as recommended by the manufacturer. The purified DNA was finally eluted in 20 μL water. The remaining 1 μL of digested DNA was diluted tenfold in water and used as input controls in subsequent amplifications.

To detect gene-specific methylation, the isothermal recombinase polymerase amplification [35] (RPA, TwistDx, UK) was employed to amplify the GSTP1 locus with primers described above in a modified RPA protocol. Briefly, 1 μL of gDNA from the above step was used for each 12.5 μL RPA reaction supplemented with 10 μM biotin-14-dUTP and 7 mM MgOAc at 37 °C for 15 min. After amplification, 12.5 μL of Agencourt AMPure XP bead solution was used to remove excess biotin and purify amplicons. The purified amplicons were eluted in 15 μL of water where 3 μL was subjected to gel electrophoresis to verify amplification.

GSTP1 primers were as described earlier. ESR1 forward and reverse primers were GTTCGTCCTGGGACTGCACTTGCTCCCGTC and AGATGCTTTGGTGTGGAGGGTCATGGTCATGGT, respectively.

To detect amplicons colorimetrically, 1 μL of purified amplicons was reacted with 20 μL SA-HRP (1/2000 dilution in 1× PBS) and SA magnetic beads (1/20 dilution in 1× PBS, NEB) for 5 mins. After collecting DNA-bound beads with a magnet and three 1× PBS washes, 50 μL of TMB substrate solution was allowed to react with the captured HRP and absorbance readings were taken after a 15-min incubation.

### Electrochemical assay

To enable an electrochemical readout, 50 μL of 0.5 M H_2_SO_4_ was used to stop the HRP/TMB reaction after 15 mins and to convert TMB to its electrochemically active form [21]. Fifty microlitres of the resulting TMB solution was added onto screen-printed electrodes (CH Instruments, USA) and electrochemical response was detected using ampometry at 150 mV over 30s on a potentiostat (CH Instruments, USA). Current response at 10 s was used for subsequent analysis.

## Additional file

Additional file 1:
**Colorimetric detection of both total genomic and loci specific DNA methylation from limited DNA inputs. Figure S1.** Total genomic methylation assay. **Figure S2.** Total genomic assay specificity and variability assessment. **Figure S3.** Effect of MBD incubation time on enrichment performance. **Figure S4.** Gene specific assay variability assessment. **Figure S5.** Detecting methylation at the ESR1 gene. **Figure S6.** Representative ampometry profiles of the four prostate cancer samples (P1 to P4) used in this study.
