# Supplementary material for: Colorimetric detection of both total genomic and loci-specific DNA methylation from limited DNA inputs
Source: Clin Epigenetics. 2015 Jul 11;7(1):65. doi: 10.1186/s13148-015-0100-6 (PMC4498563; doi:10.1186/s13148-015-0100-6)
Supplement: Additional file 1: — Colorimetric detection of both total genomic and loci specific DNA methylation from limited DNA inputs. Figure S1. Total genomic methylation assay. Figure S2. Total genomic assay specificity and variability assessment. Figure S3. Effect of MBD incubation time on enrichment performance. Figure S4. Gene specific assay variability assessment. Figure S5. Detecting methylation at the ESR1 gene. Figure S6. Representative ampometry profiles of the four prostate cancer samples (P1 to P4) used in this study. [file 13148_2015_100_MOESM1_ESM.docx]

**Supplementary Data for**

**Colorimetric detection of both total genomic and loci specific DNA methylation from limited DNA inputs**

**Fig S1. Total genomic methylation assay.** Representative absorbance of HRP/TMB reaction over time for titration over a range %methylation gDNA input.

**Fig S2. Total genomic assay specificity and variability assessment.** HRP/TMB response of a 114 bp synthetic dsDNA where all cytosines were either hydroxymethylated (hmC), methylated (5mC) or unmethylated (C) repeated over 3 independent runs performed on different days. Error bars represent SD, n = 2. Data suggests that the assay is highly reproducible and was highly specific only to 5mC methylated DNA under the current assay conditions.

**Fig S3. Effect of MBD incubation time on enrichment performance.** Representive gel electrophoresis image of an RPA using GSTP1 primers. Using M-WGA, enrichment performance was evaluated as function of MBD incubation time (10 - 25 minutes) and assesed by the yield of RPA amplicons. From as quick as a 10 min incubation resulted in similar yields as 15, 20 and 25 mins. With the goal of an rapid assay and for technical convienece when handling multiple samples, we decided on a 15 minute MBD incubation for subsequent experiments.

**Fig S4. Gene specific assay variability assessment.** HRP/TMB response of M-WGA and U-WGA over 4 independent GSTP1 assay runs performed on different days. Error bars represent SD, n = 2.

**Fig S5. Detecting methylation at the ESR1 gene.** (A) Gel electrophoresis images of RPA amplifications and photo of HRP/TMB reactions. Top: Post MBD enrichment. Bottom: input controls i.e. before MBD enrichment. (B) Corresponding absorbance measurements. Blue: post MBD enrichment. Red: input control. (C) Normalized methylation scores of HRP/TMB response. M-WGA: methylated control. WT: naïve Jurkat cells. 5-Aza: demethylating agent treated Jurkat cells. U: unmethylated control. Error bars represent SD, n = 3.

**Fig S6**. Representive ampometry profiles of the 4 prostate cancer samples (P1 to P4) used in this study. Blue: response from MBD enriched fraction. Red: input control.
